# Circulating Neprilysin Level Predicts the Risk of Cardiovascular Events in Hemodialysis Patients

**DOI:** 10.3389/fcvm.2021.684297

**Published:** 2021-06-15

**Authors:** Hyeon Seok Hwang, Jin Sug Kim, Yang Gyun Kim, Yu Ho Lee, Dong-Young Lee, Shin Young Ahn, Ju-Young Moon, Sang-Ho Lee, Gang-Jee Ko, Kyung Hwan Jeong

**Affiliations:** ^1^Division of Nephrology, Department of Internal Medicine, KyungHee University, Seoul, South Korea; ^2^Division of Nephrology, Department of Internal Medicine, CHA Bundang Medical Center, CHA University, Seongnam, South Korea; ^3^Division of Nephrology, Department of Internal Medicine, Veterans Health Service Medical Center, Seoul, South Korea; ^4^Division of Nephrology, Department of Internal Medicine, Korea University College of Medicine, Seoul, South Korea

**Keywords:** cardiovascular disease, hemodialysis, neprilysin, atherosclerosis, left ventricular systolic dysfunction

## Abstract

**Background:** Neprilysin inhibition has demonstrated impressive benefits in heart failure treatment, and is the current focus of interest in cardiovascular (CV) and kidney diseases. However, the role of circulating neprilysin as a biomarker for CV events is unclear in hemodialysis (HD) patients.

**Methods:** A total of 439 HD patients from the K-cohort were enrolled from June 2016 to April 2019. The plasma neprilysin level and echocardiographic findings at baseline were examined. The patients were prospectively followed up to assess the primary endpoint (composite of CV events and cardiac events).

**Results:** Plasma neprilysin level was positively correlated with left ventricular (LV) mass index, LV end-systolic volume, and LV end-diastolic volume. Multivariate linear regression analysis revealed that neprilysin level was negatively correlated with LV ejection fraction (β = −2.14; *p* = 0.013). The cumulative event rate of the composite of CV events was significantly greater in neprilysin tertile 3 (*p* = 0.049). Neprilysin tertile 3 was also associated with an increased cumulative event rate of cardiac events (*p* = 0.016). In Cox regression analysis, neprilysin tertile 3 was associated with a 2.61-fold risk for the composite of CV events [95% confidence interval (CI), 1.37–4.97] and a 2.72-fold risk for cardiac events (95% CI, 1.33–5.56) after adjustment for multiple variables.

**Conclusions:** Higher circulating neprilysin levels independently predicted the composite of CV events and cardiac events in HD patients. The results of this study suggest the importance of future studies on the effect of neprilysin inhibition in reducing CV events.

## Introduction

Cardiovascular (CV) disease is a major cause of death in patients undergoing hemodialysis (HD) treatment, and an extremely high rate of CV complications has been reported in these patients (1, 2). HD patients are consistently exposed to risk factors for uremia, hemodynamic overload, and sympathetic and neurohumoral activation (3, 4). Furthermore, HD treatment itself induces metabolic derangement and electrolyte shift in cardiomyocytes, episodic cardiac ischemia, and fibrosis (5, 6). Therefore, HD patients experience repetitive cardiac injuries, and these adverse processes induce cardiac dysfunction, structural changes, and remodeling, which are key factors for high CV morbidity and mortality rates.

Natriuretic peptides have been introduced into the dialysis setting, based on their pathophysiologic role in heart failure, to assess for myocardial ischemia, systolic dysfunction, and risk of future cardiac events (7, 8). Natriuretic peptides have a wide range of CV effects contributing to natriuresis, vasodilation, and blood pressure regulation. Neprilysin is the key enzyme responsible for their degradation, and its inhibition enhances the effect of natriuretic peptides on the CV system (9, 10). Previous reports have identified that neprilysin exists in soluble form in the bloodstream, and demonstrated that circulating neprilysin plays a central role in neurohormonal regulation, CV remodeling, and CV dysfunction (11–13). The neprilysin is also anticipated to be a promising biotarget for the reduction of CV risk in patients with chronic kidney disease (CKD) and cross-sectional study showed the usefulness of neprilysin for heart failure diagnosis in dialysis patients (14–18). However, almost studies exploring the neprilysin in patients with CKD enrolled the patients who were not receiving dialysis, and no evidence exists on circulating neprilysin as a pathologic surrogate to predict the incident CV events in patients undergoing HD treatment.

Therefore, we performed this study to test the hypothesis that plasma neprilysin levels are independently associated with an increased risk for future CV events in HD patients. We also investigated circulating neurohormonal markers and echocardiographic parameters to determine their relationships with neprilysin level.

## Materials and Methods

### Study Population

All data in this study were obtained from the registry of the K-cohort, which is a multicenter, prospective cohort of HD patients in Korea. The inclusion and exclusion criteria have been previously described (19). A total of 637 patients were recruited between June 2016 and April 2019, and 439 patients with whole plasma samples at the time of study enrollment were included in this study.

The study protocol was approved by the local ethics committee (KHNMC 2016-04-039), and the study was conducted in accordance with the principles of the Second Declaration of Helsinki. All participants involved in the study signed written informed consent forms before enrollment.

### Data Collection and Definitions

Demographic factors, comorbid conditions, laboratory data, dialysis information, and concomitant medication were collected at the time of inclusion. Information on patient comorbidities was derived to calculate the Charlson comorbidity index score (20). Blood samples for laboratory test and biomarkers were drawn before the start of HD in a mid-week dialysis session. Laboratory data were collected, and delivered *spKt/V* (*K*, dialyzer clearance; *t*, time; *V*, urea distribution volume) was assessed using the conventional method (21). Body mass index was calculated as body weight divided by the square of body height.

The patients were classified into three groups based on the circulating level of neprilysin: tertile 1, <107.0 pg/ml; tertile 2, 107.0–237.5 pg/ml; and tertile 3, ≥ 237.5 pg/ml. All patients were prospectively followed up after baseline assessments. The patient follow-up was censored at the time of transfer to peritoneal dialysis, kidney transplantation, follow-up loss, or patient consent withdrawal.

### Laboratory Measures

Plasma samples for neurohormonal assessment were collected using ethylenediaminetetraacetic acid-treated tubes at the time of study entry. After centrifugation for 15 min at 1000 g at room temperature, the samples were stored at −80°C until use. Enzyme-linked immunosorbent assay was performed using Magnetic Luminex® Screening Assay multiplex kits (R&D Systems Inc., Minneapolis, MN, USA) to measure B-type natriuretic peptide (BNP), N-terminal-pro-B-type natriuretic peptide (NT-proBNP), interleukin-6 (IL-6), and galectin-3. Neprilysin levels were measured using a modified sandwich immunoassay (product no. SK00724-01; Aviscera Biosciences, Santa Clara, CA, USA). All patient samples were quantified for relevant markers; however, IL-6 was only measured in a subset of patients due to sample availability. IL-6 level was measured in 331 (75.4%) patients [128 (87.1%) patients in neprilysin tertile 1, 115 (78.8%) patients in neprilysin tertile 2, and 88 (60.3%) patients in neprilysin tertile 3]. The proportion of IL-6-measured patients was significantly lower in patients with neprilysin tertile 3 (*p* < 0.001). hsCRP was measured using immunoturbidimetric method on Beckman Coulter AU5800 Analyzers (Brea, CA, USA).

### Echocardiographic Measures

Of all patients, 355 (80.1%) patients received echocardiographic examination [123 (83.7%) in tertile 1, 113 (77.4%) in tertile 2, and 119 (81.5%) in tertile 3]. The echocardiographic data was collected from clinical report. M-mode and 2D measurements were conducted by trained sonographers or cardiologist in accord with methods recommended by the American Society of Echocardiography (22). Echocardiographic examiners were blinded to the clinical data and biomarker measurements and cardiologists adjudicate and confirm all echocardiographic findings. LV end-diastolic diameter (LVDd), LV end-systolic diameter (LVDs), LV posterior wall thickness (PWT), and interventricular septal thickness (IVST) was measured in M-mode plane. LV mass was estimated using the Devereux formula and body surface area was used to index the LV mass. LV end-diastolic volume (LVEVd), LV end-systolic volume (LVEVs), LV ejection fraction (LVEF), and left atrial dimensions were determined in apical two- and four-chamber views. Peak early diastolic flow velocity (E) and peak late diastolic flow velocity (A) were determined from the mitral valve inflow velocity curve in pulsed wave Doppler. Peak early diastolic tissue velocity (E′) was measured from the septal aspect of the mitral annulus in tissue Doppler. The ratio of E to A wave (E/A) and E to E′ (E/E′) was calculated.

### Outcome Measures

The primary study endpoint was a composite of incident CV events, including cardiac and non-cardiac vascular events. Cardiac events were defined as acute coronary syndrome, heart failure, ventricular arrhythmia, cardiac arrest, and sudden death. Non-cardiac events included cerebral infarction, cerebral hemorrhage, and peripheral vascular occlusive diseases requiring revascularization or surgical intervention. All mortality events from any cause were recorded and carefully reviewed. The secondary endpoints were levels of circulating neurohormonal markers and echocardiographic parameters, and their correlations with neprilysin level were analyzed.

### Statistical Analysis

Data are expressed as mean ± standard deviation (SD) or median [interquartile range (IQR)]. Kolmogorov-Smirnov test was used to assess the normality of the distribution of the variables. Differences among the three groups were identified using analysis of variance or Kruskal-Wallis test. Tukey *post hoc* test and Mann-Whitney *U*-test with Bonferroni correction were used to identify differences between more than two groups. Categorical variables were compared using the chi-square test or Fisher's exact test. Log-transformed values of high-sensitivity C-reactive protein (hsCRP) levels were used in regression analysis because of a skewed distribution. The values of neprilysin levels were log-transformed for linear regression analysis, and 1 SD was used for hazard ratio (HR) calculations. Spearman's analyses were used to evaluate the correlation between neprilysin level and continuous variables. The association between neprilysin level and LVEF was identified using linear regression analysis. A Cox proportional hazard model was constructed to identify independent variables related to CV events or patient death. Multivariate models included significantly associated parameters according to their weight in univariate testing and clinically fundamental parameters. Baseline characteristics and laboratory data was compared between patients with and without incident cardiac event to adjust the multivariate model (Supplementary Table 1). Charlson comorbidity score, prevalence of CV event history, hemoglobin level, and plasma NT-proBNP level was significantly different between two groups. All of these parameters were included in multivariate Cox model. We tried to adjust baseline cardiac remodeling status using NT-proBNP or BNP, because echocardiographic data is not fully investigated in this study. Statistical analyses were performed using SPSS software (version 22.0; SPSS, IBM Corp., Armonk, NY, USA). *p*-values <0.05 were considered significant.

## Results

### Baseline Demographic Characteristics and Laboratory Data

The median neprilysin level was 155.2 (IQR 88.6, 304.2) pg/ml in all studied patients. According to tertile, the median neprilysin level was 68.4 (IQR 45.2, 89.2) pg/ml in tertile 1 (*n* = 147), 155.9 (IQR 127.2, 184.4) pg/ml in tertile 2 (*n* = 146), and 424.0 (IQR 303.4, 741.6) pg/ml in tertile 3 (*n* = 146). The baseline patient demographics, clinical characteristics, and laboratory results are described in Table 1. Patients in tertile 3 of neprilysin level were younger and had a shorter duration of dialysis therapy than those in neprilysin tertile 1. Twenty (4.6%) patients with heart failure were enrolled in this study. Heart failure with preserved LVEF was observed in 6 (1.4%) patients and heart failure with reduced LVEF in 14 (3.2%) patients. Laboratory data and dialysis characteristics did not show significant differences. Among the circulating neurohormonal markers, galectin-3 showed a significantly higher level in patients in neprilysin tertile 3 than in patients in the other tertiles.

**Table 1:** Baseline demographic and laboratory data of the study population.

	**Tertiles of neprilysin level**			
	**Tertile 1** ** <107.0 pg/ml** ** (*n* = 147)**	**Tertile 2 107.0–237.5 pg/ml (*n* = 146)**	**Tertile 3** ** ≥ 237.5 pg/ml** ** (*n* = 146)**	***P*-value**
Age (years)	64.9 ± 12.1	61.5 ± 11.4^*^	58.7 ± 14.2^*^	<0.001
Male (%)	109 (74.1)	92 (63.0)	91 (62.3)	0.055
Body mass index (kg/m^2^)	23.43 ± 3.82	22.99 ± 3.90	23.43 ± 4.55	0.567
HD duration (years)	4.66 ± 6.01	3.63 ± 5.07	2.91 ± 4.54^*^	0.017
Diabetes (%)	77 (52.4)	88 (60.3)	84 (57.5)	0.383
History of CV event (%)	64 (43.5)	62(42.5)	59 (40.4)	0.859
Charlson comorbidity score	4.10 ± 1.71	4.13 ± 1.21	3.97 ± 1.55	0.616
Hemoglobin (g/dl)	10.55 ± 1.28	10.46 ± 1.14	10.39 ± 1.26	0.529
Albumin (g/dl)	3.77 ± 0.33	3.82 ± 0.30	3.84 ± 0.34	0.156
LDL-cholesterol (mg/dl)	76.08 ± 25.62	77.72 ± 28.50	75.46 ± 24.84	0.752
hsCRP (mg/dl)	1.42 (0.20, 3.49)	0.92 (0.19, 3.54)	0.70 (0.19, 2.80)	0.149
Predialysis SBP (mmHg)	144.3 ± 18.9	141.3 ± 19.9	142.3 ± 21.8	0.421
Ultrafiltration (L)	2.25 ± 1.11	2.14 ± 1.07	2.29 ± 1.07	0.462
spKt/V	1.59 ± 0.46	1.55 ± 0.31	1.58 ± 0.25	0.634
ESA use (%)	131 (89.1)	132 (91.0)	136 (93.2)	0.479
BNP (pg/ml)	50.6 (16.3, 91.0)	37.3 (7.6, 86.6)	35.4 (7.6, 88.5)	0.384
NT-proBNP (pg/ml)	286 (200, 442)	330 (189, 475)	317 (198, 477)	0.542
IL-6 (pg/ml)	3.3 (2.2, 5.8)	3.0 (1.9, 4.8)	2.7 (2.1, 4.2)^*^	0.032
Galectin-3 (ng/ml)	17.2 (15.0, 19.8)	17.5 (15.0, 20.5)	19.0 (15.4, 22.0)^*^	0.029

*IL-6 was measured in 331 (75.4%) patients*.

* *p <0.05 vs. tertile 1*.

*HD, hemodialysis; CV, cardiovascular; LDL, low-density lipoprotein; hsCRP, high-sensitivity C-reactive protein; SBP, systolic blood pressure; spKt/V, single-pool Kt/V; ESA, erythropoietin-stimulating agent; BNP, brain natriuretic peptide; NT-proBNP, N-terminal-pro-B-type natriuretic peptide; MMP-2, matrix metalloproteinase-2*.

### Correlation of Neprilysin Level With Circulating Cardiac Markers and Echocardiographic Parameters

The correlations between the levels of neprilysin and circulating neurohormonal markers are shown in Table 2 and Supplementary Figure 1. The plasma levels of BNP and NT-proBNP did not show a significant correlation with neprilysin level. A significant positive correlation was found between galectin-3 and neprilysin levels, and the circulating levels of hsCRP and IL-6 were negatively correlated with neprilysin level. However, all coefficient values and distribution patterns suggested that the correlation power was not strong.

**Table 2:** Correlation of neprilysin level with circulating cardiac markers and echocardiographic parameters.

	**Correlation coefficient**	***P*-value**
**Circulating neurohormonal marker**		
BNP (pg/ml)	−0.092	0.055
NT-proBNP (pg/ml)	0.003	0.949
hsCRP (mg/dl)	−0.105	0.029
IL-6 (pg/ml)	−0.134	0.014
Galectin-3 (ng/ml)	0.124	0.009
**Echocardiographic parameters**		
LV mass index (g/m^2^)	0.129	0.043
LVDs (mm)	0.068	0.235
LVDd (mm)	0.063	0.235
LVESV (ml)	0.137	0.035
LVEDV (ml)	0.137	0.035
LVEF (%)	−0.185	<0.001
IVST (mm)	0.046	0.470
PWT (mm)	−0.023	0.690
E/E'	0.019	0.778
E/A	0.024	0.731
LA dimension (mm)	−0.048	0.445

*Echocardiography was examined in 355 (80.9%) patients*.

*BNP, B-type natriuretic peptide; NT-proBNP, N-terminal-pro-B-type natriuretic peptide; MMP-2, matrix metalloproteinase-2; LV, left ventricle; LVDs, left ventricular end-systolic diameter; LVDd, left ventricular end-diastolic diameter; LVESV, left ventricular end-systolic volume; LVEDV, left ventricular end-diastolic volume; IVST, interventricular septal thickness in diastolic; PWT LV, posterior wall thickness in diastolic; LVEF, left ventricle ejection fraction; LA, left atrium*.

The baseline echocardiographic measurements are described in Supplementary Table 2. LVEF was significantly different across tertiles, and the lowest LVEF was observed in patients in neprilysin tertile 3. Posterior wall thickness and the E/A ratio showed different mean values among the neprilysin tertiles. To investigate the relationship between neprilysin level and cardiac structures, the correlation between neprilysin level and echocardiographic parameters was evaluated (Table 2). LV systolic and diastolic diameters, LV wall thickness, and diastolic parameters were not correlated with circulating neprilysin level. A significant negative correlation was observed between LVEF and neprilysin level. LV mass index, LVESV, and LVEDV were positively correlated with neprilysin level. However, the coefficient values and distribution patterns of variables indicated weak correlation power (Supplementary Figure 2).

### Relationship Between Plasma Neprilysin Level and Left Ventricular Ejection Fraction in Hemodialysis Patients

Univariate and multivariate linear regression models were constructed to determine the association between neprilysin level and LV systolic function. In univariate analysis, LVEF was significantly associated with history of CV events (β = −2.89; *p* = 0.001), NT-proBNP level (β = −0.01; *p* = 0.023), and neprilysin level (β = −2.19; *p* = 0.011). Hemoglobin level (β = 0.50; *p* = 0.155) and ultrafiltration volume (β = −0.65; *p* = 0.115) showed borderline significance in association with neprilysin level. The multivariate linear regression model is shown in Table 3. History of CV events (β = −2.93; *p* = 0.002) and neprilysin level (β = −2.14; *p* = 0.013) were independent determinants of LVEF in HD patients.

**Table 3:** Relationship between the baseline parameters and LVEF.

	**Unstandardized β**	**95% CI**	***P*-value**
Age (years)	0.03	−0.40, 0.10	0.417
Male	−0.57	−2.36, 1.22	0.531
History of CV event	−2.93	−4.68, −1.18	0.002
Hemoglobin (g/dl)	0.56	−0.12, 1.24	0.107
NT-proBNP (pg/ml)	−0.003	−0.007, 0.001	0.112
Ultrafiltration (L)	−0.54	−1.35, 0.27	0.193
Neprilysin (pg/ml)	−2.14	−3.83, −0.45	0.013

*Of the all studied patients, 355 (80.9%) patients were examined with echocardiography. CV, cardiovascular; NT-proBNP, N-terminal-pro-B-type natriuretic peptide*.

### Prognostic Utility of Neprilysin Level in Hemodialysis Patients

**Figure 1:**
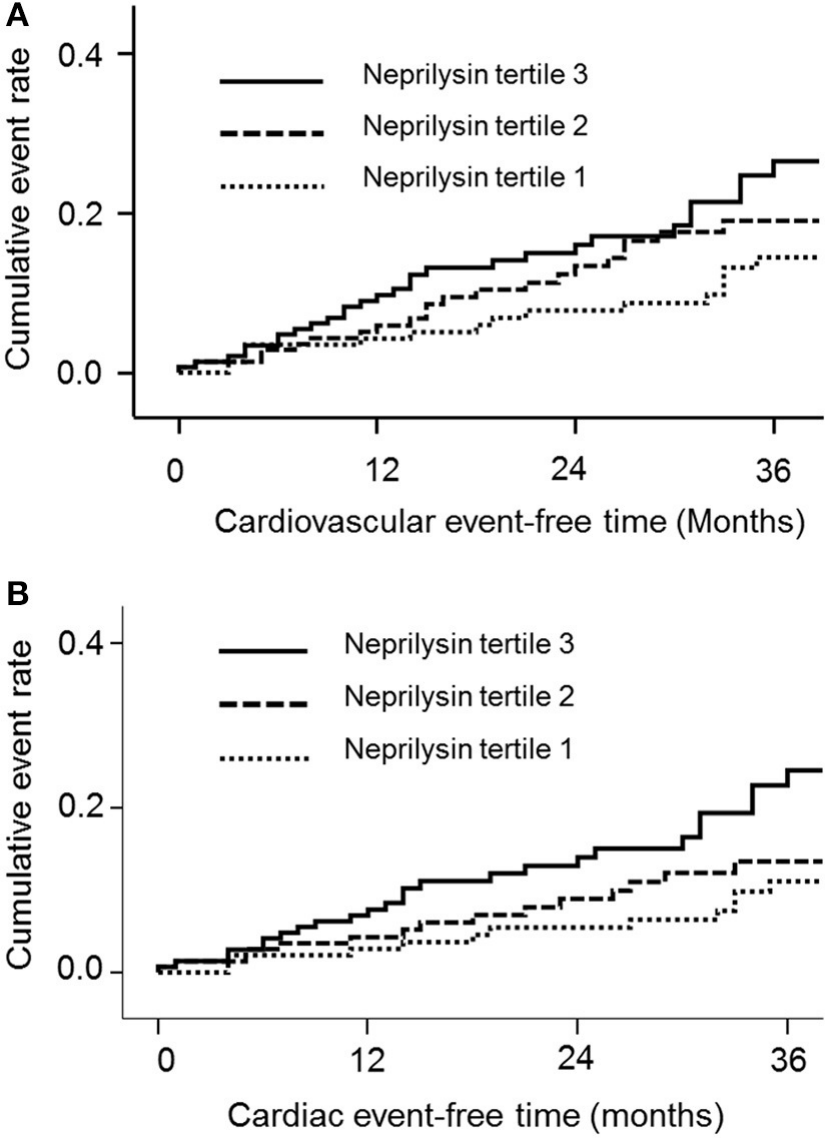
Cumulative event rates of the composite of **(A)** cardiovascular events and **(B)** cardiac events according to neprilysin level.

During a mean follow-up of 30.1 months, 61 deaths (13.9%) and 66 CV events (15.0%) occurred. Of the CV events, acute coronary syndrome occurred in 27 patients, heart failure occurred in 6 patients, ventricular arrhythmia occurred in 4 patients, cardiac arrest occurred in 9 patients, sudden death occurred in 6 patients, cerebral vascular accidents occurred in 8 patients, and peripheral vascular occlusive diseases occurred in 6 patients. The cumulative event rate of the composite of CV events was significantly greater in neprilysin tertile 3 (*p* = 0.049; Figure 1A). Neprilysin tertile 3 was associated with a greater cumulative event rate of cardiac events (*p* = 0.016; Figure 1B). The cumulative event rate of patient death did not differ among patients in the different neprilysin tertiles (*p* = 0.127).

Univariable Cox regression analysis revealed that plasma neprilysin tertile 3 was significantly associated with an increased risk for the composite of CV events [HR, 2.10; 95% confidence interval (CI), 1.14–3.88; *p* = 0.017; Table 4]. This association remained significant after adjustment for multiple variables (HR, 2.61; 95% CI, 1.37–4.97; *p* = 0.004). Neprilysin increment per 1 SD also had an independent risk for composite events (HR, 1.40; 95% CI, 1.17–1.66; *p* < 0.001). To further investigate the risk for the composite of CV events, the HRs for cardiac events and non-cardiac vascular events were evaluated. Patients in neprilysin tertile 3 had a significant risk for cardiac events after adjustment for multiple covariates (HR, 2.72; 95% CI, 1.33–5.56; *p* = 0.006) and neprilysin increment per 1 SD was also associated with the risk of cardiac events (HR, 1.44; 95% CI, 1.20–1.74; *p* < 0.001). However, neprilysin level per tertile or per 1 SD increment did not show a significant risk for non-cardiac events and patient death. We re-construct multivariate Cox hazard model including BNP as covariates, instead of NT-proBNP. Neprilysin tertile 3 and neprilysin increment per 1 SD was significantly associated with higher risk of CV composite and cardiac events (Supplementary Table 3).

**Table 4:** Hazard ratios of neprilysin tertiles for cardiovascular events.

	**No. event (%)**	**HR (95% CI), crude**	**HR (95% CI), adjusted**
**Composite of CVE**			
Neprilysin tertile 1	16 (10.9)	Reference	Reference
Neprilysin tertile 2	21 (14.4)	1.46 (0.76, 2.81)	1.77 (0.90, 3.49)
Neprilysin tertile 3	29 (19.9)	2.10^*^ (1.14, 3.88)	2.61^*^ (1.37, 4.97)
Neprilysin per SD		1.32^*^ (1.11, 1.57)	1.40^*^ (1.17, 1.66)
**Cardiac event**			
Neprilysin tertile 1	12 (8.2)	Reference	Reference
Neprilysin tertile 2	16 (11.0)	1.45 (0.70, 2.98)	1.56 (0.72, 3.36)
Neprilysin tertile 3	26 (17.8)	2.54^*^ (1.28, 5.05)	2.72^*^ (1.33, 5.56)
Neprilysin per SD		1.37^*^ (1.14, 1.64)	1.44^*^ (1.20, 1.74)
**Non-cardiac vascular event**			
Neprilysin tertile 1	4 (2.7)	Reference	Reference
Neprilysin tertile 2	7 (4.8)	1.84 (0.54, 6.27)	2.56 (0.68, 9.72)
Neprilysin tertile 3	4 (2.7)	1.00 (0.25, 4.02)	1.47 (0.34, 6.30)
Neprilysin per SD		1.26 (0.89, 1.78)	1.27 (0.90, 1.78)
**Patient death**			
Neprilysin tertile 1	29 (19.7)	Reference	Reference
Neprilysin tertile 2	16 (11.0)	0.59 (0.32, 1.09)	0.75 (0.40, 1.42)
Neprilysin tertile 3	16 (11.0)	0.60 (0.33, 1.11)	0.81 (0.43, 1.55)
Neprilysin per SD		0.81 (0.52, 1.26)	0.88 (0.60, 1.29)

*All analyses are adjusted for the following: age, sex, body mass index, Charlson comorbidity index, hemoglobin, hsCRP, NT-proBNP, ESA use, HD duration, spKt/V*.

*hsCRP, high-sensitivity C-reactive protein; NT-proBNP, N-terminal-pro-B-type natriuretic peptide; ESA, erythropoietin-stimulating agent; HD, hemodialysis; spKt/V, single-pool Kt/V*.

* *p <0.05*.

## Discussion

Our prospective observational cohort study demonstrated that an increased level of neprilysin was associated with a greater cumulative event rate of CV composites and cardiac events. In addition, higher levels of neprilysin increased the risk for incident CV composites and cardiac events after adjustment for multiple covariates. Plasma neprilysin level was positively correlated with galectin-3 circulating level, LV dimension, and LV mass index. In addition, an independent negative relationship was observed between neprilysin level and LVEF. These findings suggest that neprilysin is a novel biomarker for assessing the risk of CV events, and that it is associated with cardiac structural and functional changes in HD patients.

Interestingly, we found a weakly negative correlation between neprilysin and the inflammatory markers hsCRP and IL-6. Inflammatory substrates are known to be degradable by neprilysin, and we presumed that higher levels of neprilysin are associated with a reduced inflammatory state. However, the correlation power between neprilysin and inflammatory marker was weak, suggesting that the inhibitory interaction was not substantial in HD patients (9, 23). The additional incidental finding was negative correlation between neprilysin levels and age (ρ = −0.167; *p* < 0.001). The reason of this finding is not clear, but we presumed that the correlation between circulating neprlysin level and age is dependent on population characteristics, because the correlations coefficients were changeable in different patient categories (11, 24, 25).

Galectin-3 is a contributing factor to cardiac fibrosis, and a biomarker for LV remodeling and heart failure progression (26–28). Our results showed that neprilysin level was positively correlated with galectin-3 level. In addition, LV internal volume and LV mass index increased as the plasma level of neprilysin increased. These findings suggest that circulating neprilysin level reflects the pathologic deformation of cardiac structures. However, correlation power indicated the weak relationship between neprilysin and echocardiographic parameters. Therefore, we construct linear regression model to find out independent relationship between neprilysin and LVEF. We observed that neprilysin level was associated with lower LVEF after multiple adjustment. These findings suggest that neprilysin is a noticeable indicator of LV systolic dysfunction and cardiac remodeling in HD patients.

The use of cardiac biomarkers in clinical practice allows clinicians to identify high-risk patients for incident CV events. Although BNP and NT-proBNP have been widely used in patients with heart failure, their use in HD patients is challenging because of high individual variations, increased plasma levels without any evidence of cardiac disease, more than normal values in 90% of HD patients, and wide differences in cutoff value for risk stratification in diverse studies (29–31). Therefore, alternative cardiac biomarkers are required in dialysis care, and neprilysin is of particular interest because it is a new biotarget for innovative therapeutic strategies in heart failure (10, 32). Our study revealed that plasma neprilysin levels were significantly associated with increased rates of CV composites and cardiac events. The association remained significant after adjustment for multiple established CV risk factors, including NT-proBNP. These findings suggest that higher neprilysin levels contribute to incident CV risk independently of traditional CV risk factors and that neprilysin is a valuable biomarker for CV risk prediction in patients undergoing HD treatment.

Although we found a significant predictive ability of neprilysin for adverse CV outcomes in HD patients, recent studies on non-dialysis-dependent CKD revealed that high neprilysin levels did not predict poor CV outcomes (33). We presumed that these divergent results might originate from the greater activation of natriuretic peptide systems in HD patients. Because HD patients usually have higher degrees of cardiac remodeling than non-dialysis-dependent CKD patients, the activation of natriuretic peptides is more pronounced in HD patients (34–36). Therefore, the clinical importance of neprilysin in HD patients may become larger with activated natriuretic peptides. This explanatory assumption is also supported by the discrepant results in different heart failure settings. Previous studies have reported that circulating neprilysin level is predictive of CV death in patients with heart failure with acute decompensation or reduced LVEF, but is not associated with CV outcomes in patients with heart failure with preserved LVEF (11, 12, 37, 38).

This study had some limitations. Echocardiographic parameters and IL-6 levels were not measured in some patients. The lower proportion of IL-6-measured patients in neprilysin tertile 3 may be possible to cause the bias in correlation analysis. Furthermore, given the limited number of events, we could not perform individual analyses for heart failure, although HD patients are at a higher risk for congestion (39). In addition, we measured neprilysin concentration only, and neprilysin activity was not measured. It was reported in a previous study that neprilysin activity, but not concentration, provided diagnostic information about heart failure in dialysis patients (18). Lower neprilysin activity combining with multi-markers helped to determine the presence of heart failure. Therefore, measurement of neprilysin activity might provide additional data on the risk of incident CV event. Further studies with neurohormonal peptides, neprilysin concentration and activity may improve predictability of CV complication in HD patients.

## Conclusion

Circulating neprilysin level was correlated with pathologic remodeling of echocardiographic structures and independently associated with lower LVEF. Higher circulating neprilysin levels were associated with a greater risk of the composite of CV events and cardiac events in HD patients. Our results suggest the importance of future studies on the implication of neprilysin inhibition for HD patients.

## Data Availability Statement

The raw data supporting the conclusions of this article will be made available by the authors, without undue reservation.

## Ethics Statement

The studies involving human participants were reviewed and approved by KyungHee University Hospital IRB. The patients/participants provided their written informed consent to participate in this study.

## Author Contributions

HSH conceived the research question conceived and designed the analysis. JSK, YGK, YHL, D-YL, J-YM, JYM, and S-HL undertook data collection conducted the study. HSH, G-JK, and KHJ drafted the manuscript. All authors reviewed the results and commented on the manuscript. All authors read and approved the final manuscript.

## Conflict of Interest

The authors declare that the research was conducted in the absence of any commercial or financial relationships that could be construed as a potential conflict of interest.
