# Supplementary material for: Circulating Neprilysin Level Predicts the Risk of Cardiovascular Events in Hemodialysis Patients
Source: Front Cardiovasc Med. 2021 Jun 15;8:684297. doi: 10.3389/fcvm.2021.684297 (PMC8239158; doi:10.3389/fcvm.2021.684297)
Supplement: Supplementary file 1 [file Data_Sheet_1.docx]

Supplementary Material

**Supplementary table 1** Comparison of baseline demographic and laboratory data between patients with and without incident CV event

|  | | **Patients with cardiac event**  **(n = 54)** | **Patients without cardiac event**  **(n = 385)** | ***P* value** |
| --- | --- | --- | --- | --- |
| Age (years) | 64.0±12.6 | | 61.4±12.8 | 0.165 |
| Male (%) | 33 (61.1) | | 259 (67.3) | 0.369 |
| Body mass index (kg/m^2^) | 23.02±3.64 | | 23.32±4.16 | 0.615 |
| HD duration (years) | 3.96±4.82 | | 3.70±5.35 | 0.736 |
| History of CV event (%) | 35 (64.8) | | 150 (39.0) | <0.001 |
| Charlson comorbidity score | 4.70±1.67 | | 3.97±1.46 | 0.001 |
| Hemoglobin (g/dL) | 10.22±1.10 | | 10.51±1.24 | 0.040 |
| LDL-cholesterol (mg/dL) | 77.63±28.21 | | 76.53±26.08 | 0.814 |
| hsCRP (mg/dL) | 0.75 (0.16, 3.05) | | 0.86 (0.18, 3.16) | 0.951 |
| Predialysis SBP (mmHg) | 146.2±18.6 | | 142.1±20.4 | 0.163 |
| Ultrafiltration (L) | 2.29±1.04 | | 2.22±1.09 | 0.626 |
| spKt/V | 1.56±0.30 | | 1.58±0.36 | 0.682 |
| ESA use (%) | 48 (88.9) | | 351 (91.4) | 0.543 |
| BNP (pg/mL) | 50.6 (13.4, 178.6) | | 41.5 (13.4, 86.9) | 0.346 |
| NT-proBNP (pg/mL) | 395 (246, 523) | | 332 (202, 467) | 0.049 |
| IL-6 (pg/ml) | 3.0 (2.1, 4.8) | | 3.4 (2.2, 5.8) | 0.698 |
| Galectin-3 (ng/ml) | 17.4 (15.0, 20.8) | | 18.4 (15.6, 21.3) | 0.176 |

*IL-6 was measured in 355 (75.4%) patients.*

*HD, hemodialysis; CV, cardiovascular; LDL, low-density lipoprotein; hsCRP, high-sensitivity C-reactive protein; SBP, systolic blood pressure; spKt/V, single-pool Kt/V; ESA, erythropoietin-stimulating agent; BNP, brain natriuretic peptide; NT-proBNP, N-terminal-pro-B-type natriuretic peptide; MMP-2, matrix metalloproteinase-2;*

**Supplementary table 2** Baseline echocardiographic data of the study population

|  | **Tertiles of neprilysin level** | | |  |
| --- | --- | --- | --- | --- |
|  | **Tertile 1**  **(n = 123)** | **Tertile 2**  **(n = 113)** | **Tertile 3**  **(n = 119)** | ***P* value** |
| LV mass index (g/m^2^) | 113.4±36.8 | 121.7±33.2 | 122.1±35.2 | 0.108 |
| LVDs (mm) | 33.5±8.7 | 33.5±5.2 | 34.8±9.0 | 0.428 |
| LVDd (mm) | 50.7±7.5 | 51.7±5.5 | 51.8±5.7 | 0.404 |
| LVESV (mL) | 36.2±19.1 | 39.1±17.3 | 41.2±22.6 | 0.163 |
| LVEDV (mL) | 93.6±35.1 | 98.3±33.0 | 101.7±33.5 | 0.153 |
| LVEF (%) | 63.8±8.4 | 62.4±6.9 | 61.4±8.5 | 0.016 |
| IVST (mm) | 13.8±2.2 | 13.8±2.3 | 14.3±3.2 | 0.758 |
| PWT (mm) | 10.4±3.5 | 10.1±1.8 | 10.0±1.8 | 0.034 |
| E wave (cm/sec) | 75.1±29.1 | 78.1±22.8 | 85.0±32.4 | 0.104 |
| A wave (cm/sec) | 88.3±24.6 | 93.7±20.5 | 87.0±23.6 | 0.138 |
| E’ (cm/sec) | 5.9±1.8 | 6.1±1.7 | 6.4±1.8 | 0.117 |
| E/A | 1.04±1.57 | 0.84±0.24 | 1.01±0.51 | 0.014 |
| E/E’ | 13.3±5.3 | 13.3±4.1 | 14.2±6.9 | 0.934 |
| LA dimension (mm) | 41.3±5.5 | 41.1±6.8 | 41.0±7.8 | 0.870 |

*Echocardiography was examined in 335 (80.1%) patients.*

*LV, left ventricle; LVDs, left ventricular end-systolic diameter; LVDd, left ventricular end-diastolic diameter; LVESV, left ventricular end-systolic volume; LVEDV, left ventricular end-diastolic volume; IVST, interventricular septal thickness in diastolic; PWT, left ventriclar posterior wall thickness in diastolic; LVEF, left ventricle ejection fraction; LA, left atrium*

**Supplementary table 3** Hazard ratios of neprilysin tertiles for cardiovascular events

|  | | | **HR (95% CI), adjusted** | |
| --- | --- | --- | --- | --- |
| **Composite of CVE** |  | | |  |
| Neprilysin tertile 1 | | | Reference | |
| Neprilysin tertile 2 | | | 1.73 (0.88, 3.42) | |
| Neprilysin tertile 3 | | | 2.57* (1.36, 4.88) | |
| Neprilysin per SD | | | 1.40* (1.18, 1.67) | |
| **Cardiac event** | | |  | |
| Neprilysin tertile 1 | | | Reference | |
| Neprilysin tertile 2 | | | 1.52 (0.70, 3.29) | |
| Neprilysin tertile 3 | | | 2.74* (1.34, 5.59) | |
| Neprilysin per SD | | | 1.44* (1.20, 1.73) | |
| **Noncardiac vascular event** | |  |  |  |
| Neprilysin tertile 1 | | | Reference | |
| Neprilysin tertile 2 | | | 2.33 (0.63, 8.62) | |
| Neprilysin tertile 3 | | | 1.31 (0.31, 5.59) | |
| Neprilysin per SD | | | 1.29 (0.91, 1.82) | |
| **Patient death** | |  |  |  |
| Neprilysin tertile 1 | | | Reference | |
| Neprilysin tertile 2 | | | 0.75 (0.39, 1.42) | |
| Neprilysin tertile 3 | | | 0.86 (0.45, 1.62) | |
| Neprilysin per SD | | | 0.89 (0.62, 1.29) | |

*All analyses are adjusted for the following: age, sex, body mass index, Charlson comorbidity index, hemoglobin, hsCRP, BNP, ESA use, HD duration, spKt/V*

*hsCRP, high-sensitivity C-reactive protein; NT-proBNP, N-terminal-pro-B-type natriuretic peptide; ESA, erythropoietin-stimulating agent; HD, hemodialysis; spKt/V, single-pool Kt/V*

*^*^ p < 0.05*

**Supplementary figure 1** Correlation of neprilysin level with circulating cardiac markers

**Supplementary figure 2** Correlation of neprilysin level with echocardiographic parameters
